# Hypertension-Related Drug Activity Identification Based on Novel Ensemble Method

**DOI:** 10.3389/fgene.2021.768747

**Published:** 2021-10-15

**Authors:** Bin Yang, Wenzheng Bao, Jinglong Wang

**Affiliations:** ^1^ School of Information Science and Engineering, Zaozhuang University, Zaozhuang, China; ^2^ School of Information and Electrical Engineering, Xuzhou University of Technology, Xuzhou, China; ^3^ College of Food Science and Pharmaceutical Engineering, Zaozhuang University, Zaozhuang, China

**Keywords:** hypertension, flexible neural tree, ensemble, network pharmacology, machine learning

## Abstract

Hypertension is a chronic disease and major risk factor for cardiovascular and cerebrovascular diseases that often leads to damage to target organs. The prevention and treatment of hypertension is crucially important for human health. In this paper, a novel ensemble method based on a flexible neural tree (FNT) is proposed to identify hypertension-related active compounds. In the ensemble method, the base classifiers are Multi-Grained Cascade Forest (gcForest), support vector machines (SVM), random forest (RF), AdaBoost, decision tree (DT), Gradient Boosting Decision Tree (GBDT), KNN, logical regression, and naïve Bayes (NB). The classification results of nine classifiers are utilized as the input vector of FNT, which is utilized as a nonlinear ensemble method to identify hypertension-related drug compounds. The experiment data are extracted from hypertension-unrelated and hypertension-related compounds collected from the up-to-date literature. The results reveal that our proposed ensemble method performs better than other single classifiers in terms of ROC curve, AUC, TPR, FRP, Precision, Specificity, and F1. Our proposed method is also compared with the averaged and voting ensemble methods. The results reveal that our method could identify hypertension-related compounds more accurately than two classical ensemble methods.

## Introduction

Hypertensive disease is a frequent cardiovascular disease characterized by elevated arterial blood pressure and accompanied by the target organ injury or clinical diseases (Essiarab et al., 2011; Owlia and Bangalore, 2016). It is a risk factor leading to many serious complications such as stroke, hypertensive heart disease, renal failure, atherosclerosis, and so on (Sakai and Sigmund, 2005; Brinks and Eckhart, 2010). Due to the increasing pressure of work and life, many people do not develop good eating and living habits, and often stay up late. The age of hypertensive patients tends to be younger. Therefore, the prevention and treatment of hypertension has become very important for human health.

Network pharmacology (NP) could construct a multi-dimensional network based on “traditional Chinese medicine prescription-chemical component-targets-disease targets” to analyze the relationships between traditional Chinese medicine multi-components and activity, which could provide a theoretical basis for further experimental research on a pharmacodynamic material basis and action mechanism (Wang et al., 2018; Xu et al., 2018). In recent years, network pharmacology has revealed therapeutic targets for hypertension and become a research hotspot, as it has been clinically verified to be an effective method of drug screening (Chen et al., 2020). Chen et al. screened out the key compounds and targets of JiaWeiSiWu granule to reveal the mechanism of JiaWeiSiWu granule in treating hypertension by NP method (Chen et al., 2021a). By NP and molecular docking (MD) methods, Zhai et al. investigated the mechanism of Pinellia ternate in treating hypertension (Zhai et al., 2021). Chen et al. analyzed the network based on Guizhi decoction, active compounds, and targets, and found hypertension-related targets and key pathways (Chen et al., 2021b). Chen et al. utilized NP and MD to analyzed the genistein for treating pulmonary hypertension (PH) and provided new guidance for further PH-related research (Chen et al., 2019). Liu et al. explained the pharmacological mechanism of TaohongSiwu decoction in the treatment of essential hypertension (EH) by the NP method (Liu et al., 2020). Wang et al. utilized NP to analyze the mechanism of Yeju Jiangya decoction against hypertension (Wang et al., 2021).

In recent decades, many data mining methods have been applied to reveal the disease mechanism and medication law of many complex diseases, especially hypertension (Ji and Wang, 2014; Ji et al., 2015; Hwang et al., 2016; Hu et al., 2018; Liang et al., 2018; Amaratunga et al., 2020; Liu et al., 2021; Zhao et al., 2021). Zhang et al. utilized SPSS21.0 and Apriori algorithm to analyze the symptom/sign information of EH patients collected and gave their distribution law and correlation (Zhang et al., 2019a). Yuan and Chen proposed niche technology and an artificial bee colony algorithm to mine association rules from Traditional Chinese Medicine (TCM) cases for treating hypertension (Yuan and Chen, 2011). Ma et al. collected the new literature about hypertension and constructed the gene network by analysis (Ma et al., 2018). Ramezankhani et al. utilized a decision tree to predict the risk factors of hypertension incidence in data collected from Iranian adults (Ramezankhani et al., 2016). Aljumah et al. utilized a data mining method to predict the treatment of hypertension patients with different age groups (Aljumah et al., 2011). Fang et al. proposed a new model-based KNN and LightGBM to predict the risk of hypertension (Fang et al., 2021).

Few studies have involved the use of data mining methods to improve network pharmacology. In this paper, a novel ensemble method based on a flexible neural tree (FNT) is proposed to identify hypertension-related active compounds. In the ensemble method, the used base classifiers are Multi-Grained Cascade Forest, support vector machines, random forest, AdaBoost, decision tree, Gradient Boosting Decision Tree, KNN, logical regression, and naïve Bayes. The classification results of nine classifiers are input to the FNT model, which is trained to predict hypertension-related compounds. The data used in the experiment are from up-to-date literature collected about hypertension and network pharmacology. By analysis of the literature, hypertension-related compounds were collected as positive samples and the generated decoys were utilized as negative samples. The molecular descriptor of each compound is extracted as the feature vector.

## Methods

### Classifiers

Assume that the training data is $T=\left\{\left(x_{1},y_{1}\right),\left(x_{2},y_{2}\right),\ldots ,\left(x_{n},y_{n}\right)\right\}$ containing n sample points. Sample point $x_{i}=\left\{x_{i}^{1},x_{i}^{2},\ldots x_{i}^{m}\right\}$ contains m features and category label $y_{i}=\left\{c_{1},c_{2}\right\}$ contains two cases. The nine classifiers used are introduced in the following sections of the article.

#### Multi-Grained Cascade Forest

**FIGURE 1:**
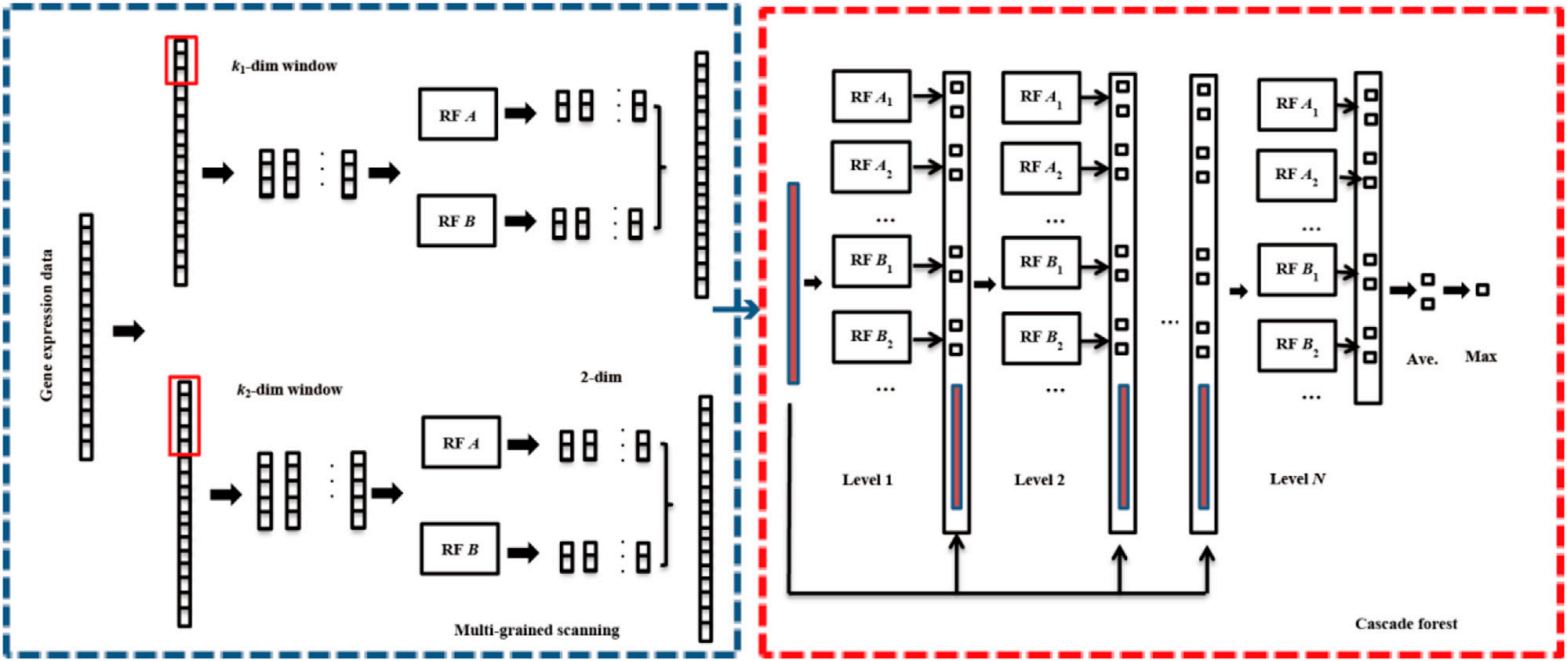
The process of gcForest.

Multi-Grained Cascade Forest (gcForest) is a novel ensemble machine learning method, which utilizes the cascade forest (ensemble of decision trees) to learn and generate models (Zhou and Feng, 2017). The core of gcForest mainly includes two modules: multi-grained scanning and cascade forest. The flowchart of gcForest is depicted in Figure 1.

1) Multi-grained scanning

Multi granularity scanning is a technical means to enhance cascade forest and do more processing on features. Firstly, a complete m - dimensional sample is input, and then sliding sampling is carried out through the $k_{1}$ -dimensional and $k_{2}$ -dimensional sampling windows in order to obtain $s_{1}=\left(m-k_{1}\right)+1$ and $s_{2}=\left(m-k_{2}\right)+1$ feature subsample vectors, respectively. Each sub-sample is used for the training of completely random forest ( A ) and random forest ( B ). A probability vector with 2-dimension is obtained in each forest, so that two kinds of forests can produce $2s_{1}$ and $2s_{2}$ representation vectors, respectively. Finally, the results of all forests are spliced together to obtain the sample output.

2) Cascade forest

Cascade forest includes several layers, each layer is composed of many forests, and each forest is composed of many decision trees. Completely random forest ( A ) and random forest ( B ) in each layer ensure the diversity of the model. For a completely random forest, each tree in the forest randomly selects a feature as the splitting node of the splitting tree, which grows until each leaf node is subdivided into only one class. For random forest, each tree randomly selects $\sqrt{m}$ candidate features, and the splitting nodes are filtered through the Gini coefficient. Each forest could generate a two-dimensional class vector. The two-dimensional class vectors of all forests are averaged to obtain the final two-dimensional class vector. Finally, the category with the maximum value in the final two-dimensional class vector is taken as the final classification result.

#### Support Vector Machines

Support vector machines (SVM) is a supervised learning algorithm based on statistical learning theory (Suykens and Vandewalle, 1999; Furey et al., 2000). With the sample set containing positive and negative samples, SVM could search a hyperplane that could segment the samples according to positive and negative classes. The classification hyperplane can be given as follows.

$$w^{T}x+b=0.$$ (1)

Where x is the data point on the classification hyperplane, w is a vector perpendicular to the classification hyperplane, and b is the displacement.

Linear separated data can be distinguished by the optimal classification hyperplane. For non-linear separated data, SVM can be transformed into solving the following optimization problem by the soft interval optimization and kernel techniques.

$$\left\{ \begin{array}{l} \min\phi \left(w,\varsigma \right)={\left\|w\right\|}^{2}+\frac{1}{2}C\sum_{i=1}^{n}\varsigma_{i}\\ s.t.y_{i}\left[\left(w\cdot x_{i}+b\right)\right]\geq 1-\varsigma_{i} \end{array}\right..$$ (2)

Where C is the penalty factor, $\varsigma_{i}$ is the relaxation variable, and $x_{i}$ is mapped to a high-dimensional space by ϕ . SVM could find a hyperplane with the largest interval in this high-dimensional space to classify the data.

#### Random Forest

Random forest (RF) is a machine learning method based on an ensemble of decision trees for classification and regression (Breiman, 2001; Díaz-Uriarte and Alvarez de Andrés, 2006). Random forest is a combined classification model composed of many decision tree classification models. Each decision tree has the right to vote to determine the best classification result. In random forest, firstly, K sample sets are extracted from the original training set by bootstrap sampling method, and the size of each extracted sample set is the same as that of the original training set. Then, K decision tree models are established from K sample sets, respectively. And K trees will create K classification results. The random forest integrates all the classified results by voting method, and the category with the most votes is designated as the final classification result.

#### AdaBoost

AdaBoost is a dynamic ensemble classification algorithm, which is to reasonably combine multiple weak classifiers (single-layer decision tree) to make it a strong classifier (Morra et al., 2009; Cao et al., 2013). The detailed algorithm is given as follows.

1) Initialize the weight of each sample. Assuming that the dataset contains n samples, each training sample point is given the same weight ( $\frac{1}{n}$ ) at the beginning.
2) Train weak classifiers. According to the samples, the weak classifiers are trained. If a sample has been accurately classified, its weight will be reduced in constructing the next training set. On the contrary, if a sample point is not accurately classified, its weight is increased. At the same time, according to the classification error of the weak classifier, its weight is calculated. Then, the sample set with updated weights is used to train the next classifier, and the whole training process goes on iteratively. T weak classifiers are obtained after T iterations.
3) The trained weak classifiers are combined into strong classifiers. Each weak classifier connects its respective weights through the classification function to form a strong classifier. After the training process of each weak classifier, the weight of the weak classifier with a smaller classification error rate is larger, which plays a greater decisive role in the final classification function, while the weight of the weak classifier with a larger classification error rate is smaller, which plays a smaller decisive role in the final classification function.

#### Decision Tree

A Decision Tree (DT) learning algorithm is usually a process of recursively selecting the optimal features and segmenting the training data according to the features so that each sub dataset has the best classification. The CART algorithm is one of the most common decision tree algorithms, which is mainly used for classification and regression (Breiman et al., 1984; Temkin et al., 1995). CART introduces the knowledge of probability theory and statistics into the research of decision tree. Different from the C4.5 algorithm, the CART algorithm could make a binary partition of the feature space and can split scalar attributes and continuous attributes. The specific algorithm is as follows:

1) Calculate the Gini index of the existing features. The feature with the smallest Gini index is selected as the splitting attribute of the root node. According to the optimal feature and cut point, two sub-nodes are generated from the current node, and the training dataset is allocated to the two sub-nodes according to the feature. According to an attribute value, a node is segmented to make the data in each descendant subset more “pure” than the data in its parent subset. Gini coefficient measures the impurity of sample division, and the smaller the impurity is, the higher the “purity” of the samples is.

For 2-class problems, the training set S is divided into two subsets $S_{1}$ and $S_{2}$ according to an attribute A . The Gini coefficient of the given division S is calculated as follows.

$$Gini_{A}\left(S\right)=\frac{\left|S_{1}\right|}{\left|S\right|}Gini\left(S_{1}\right)+\frac{\left|S_{2}\right|}{\left|S\right|}Gini\left(S_{2}\right).$$ (3)

Where |S| is the number of samples in set S , and Gini(S)i is the Gini coefficient of sample set $S_{i}$ , which is calculated as follows:

$$Gini\left(S_{i}\right)=1-{\sum_{k=1}^{2}\left(\frac{\left|C_{k}\right|}{\left|S_{i}\right|}\right)}^{2}.$$ (4)

Where $\left|C_{k}\right|$ denotes the number of samples belonging to class *k* in the set $S_{i}$ .

2) Step (1) is called recursively for two child nodes, and the iteration continues until the samples in all child nodes belong to the same category or no attributes can be selected as splitting attributes.
4) Prune the CART decision tree generated.

#### Gradient Boosting Decision Tree

Gradient Boosting Decision Tree (GBDT) is an integrated learning algorithm (Hu and Min, 2018; Zhang et al., 2019b). By boosting method, N weak learners are created, which are combined into a strong learner after many iterations. The performance of the strong learner is higher than any weak learner. In GBDT, the used weak learner is the CART regression tree. During each iteration of GBDT, the residual of the previous model is reduced, and a new model is trained and established in the gradient direction of residual reduction, to improve the performance of the classifier. The specific algorithm is shown as follows:

1) Initialize the weak learner.

$$f_{0}\left(x\right)=\arg\min_{\kappa }\sum_{i=1}^{n}L\left(y_{i},\kappa \right).$$ (5)

Where L is the loss function.

2) For t−th iteration ( t=1,2,…,T )

a) For i−th sample, the residual reduction is calculated as follows.

$$r_{ti}=-{\left[\frac{\partial L\left(y_{i},f\left(x_{i}\right)\right)}{\partial f\left(x_{i}\right)}\right]}_{f\left(x\right)=f_{t-1}\left(x\right)}.$$ (6)

Where $f_{t-1}\left(x\right)$ is the classifier during the t−1−th iteration.

$$\kappa_{tj}=\arg\min_{\kappa }\sum_{x_{i}\in R_{tj}}L\left(y_{i},f_{t-1}\left(x_{i}\right)+\kappa \right).$$ (7)

Where $\kappa_{tj}$ is the value of the leaf node in the regression tree.

b) The calculated residues are used as new sample data, $\left(x_{i},r_{ti}\right)$ is utilized to fit a new CART regression tree and the probability of each category is calculated. The leaf node region of the CART regression tree $R_{tj}$ ( j=1,2,…,J ) is obtained. J is the number of leaf nodes of the regression tree.
c) Calculate the optimal coefficient for the leaf area, which is given as follows.
d) The strong learner is updated with Eq. 8.
$$f_{t}\left(x\right)=f_{t-1}\left(x_{i}\right)+\sum_{j=1}^{J}\kappa_{tj}I\left(x\in R_{tj}\right).$$ (8)

When $x\in R_{tj}$ is true, I is equal to 1; otherwise, it is equal to 0.

3) The final strong learner f(x) is obtained with Eq. 9.

$$f\left(x\right)=f_{0}\left(x\right)+\sum_{t=1}^{T}\sum_{j=1}^{J}c_{tj}I\left(x\in R_{tj}\right).$$ (9)

#### K-Nearest Neighbor

K-Nearest Neighbor (KNN) is a classification algorithm based on supervised learning, which is to classify the data points according to the sample set with the known categories (Liao and Vemuri, 2002). Select the K neighbors with the smallest distance from the input data in the training set, and take the category with the most times among the K neighbors as the category of the classified data point. In the KNN algorithm, the selected neighbors are objects that have been correctly classified.

In the KNN method, the most commonly used measurement of distance is the Euclidean distance. The Euclidean distance of two variables ( $x_{i}$ and $x_{j}$ ) is defined as follows.

$$D\left((x_{i},x_{j}\right)=\sqrt{\sum_{k=1}^{m}{\left(x_{i}^{k}-x_{j}^{k}\right)}^{2}}.$$ (10)

#### Logistic Regression

Logistic regression (LR) is utilized to deal with the regression problem, which obtains the minimum result of cost function by gradient descent method to obtain the better classification boundary (Maalouf, 2011; Munshi et al., 2014). LR maps the values of linear regression to the interval [0, 1] by Sigmoid function, which is defined as follows.

$$y_{i}=h_{\theta }\left(x_{i}\right)=\frac{1}{1+e^{-\theta^{T}x_{i}}}.$$ (11)

Where $\theta^{T}x_{i}=\theta_{0}+\theta_{1}x_{i}^{1}+\theta_{1}x_{i}^{2}+\ldots +\theta_{m}x_{i}^{m}$ , $\theta_{0}$ is a deviation parameter and $\theta_{i}$ represents the weight.

In order to solve the logistic regression model, the gradient descent algorithm is generally used to iteratively calculate the optimal parameters of the model.

#### Na**ï**ve Bayes

Naïve Bayes (NB) is one of the most widely utilized models in Bayesian classifiers, which is based on the assumption that the influence of an attribute value on the given class is independent of the values of other attributes (class conditional independence) (Rish, 2001; Li and Guo, 2005). The specific algorithm idea is as follows.

According to the joint probability and the prediction data x , the prediction category of x is defined as follows.

$$\arg\max p\left(y=c_{k}|x\right).$$ (12)

According to the Bayesian theorem, $p\left(y=c_{k}|x\right)$ is calculated as follows.

$$p\left(y=c_{k}|x\right)=\frac{p\left(x|y=c_{k}\right)p\left(y=c_{k}\right)}{p\left(x\right)}.$$ (13)

Since the denominator is constant for all categories, just maximize the numerator, and Eq. 12 could be defined as follows.

$$\arg\max p\left(x|y=c_{k}\right)p\left(y=c_{k}\right).$$ (14)

Because each feature attribute is conditionally independent, $p\left(x|y=c_{k}\right)$ could be calculated as follows.

$$p\left(x|y=c_{k}\right)=\prod_{i=1}^{m}p\left(x^{i}|y=c_{k}\right)$$ (15)

According to Eq. 15, Eq. 14 can be calculated as follows.

$$\arg\max p\left(y=c_{k}\right)\prod_{i=1}^{m}p\left(x^{i}|y=c_{k}\right)$$ (16)

Select the category with the largest posteriori probability as the prediction category.

### Ensemble Methods

**FIGURE 2:**
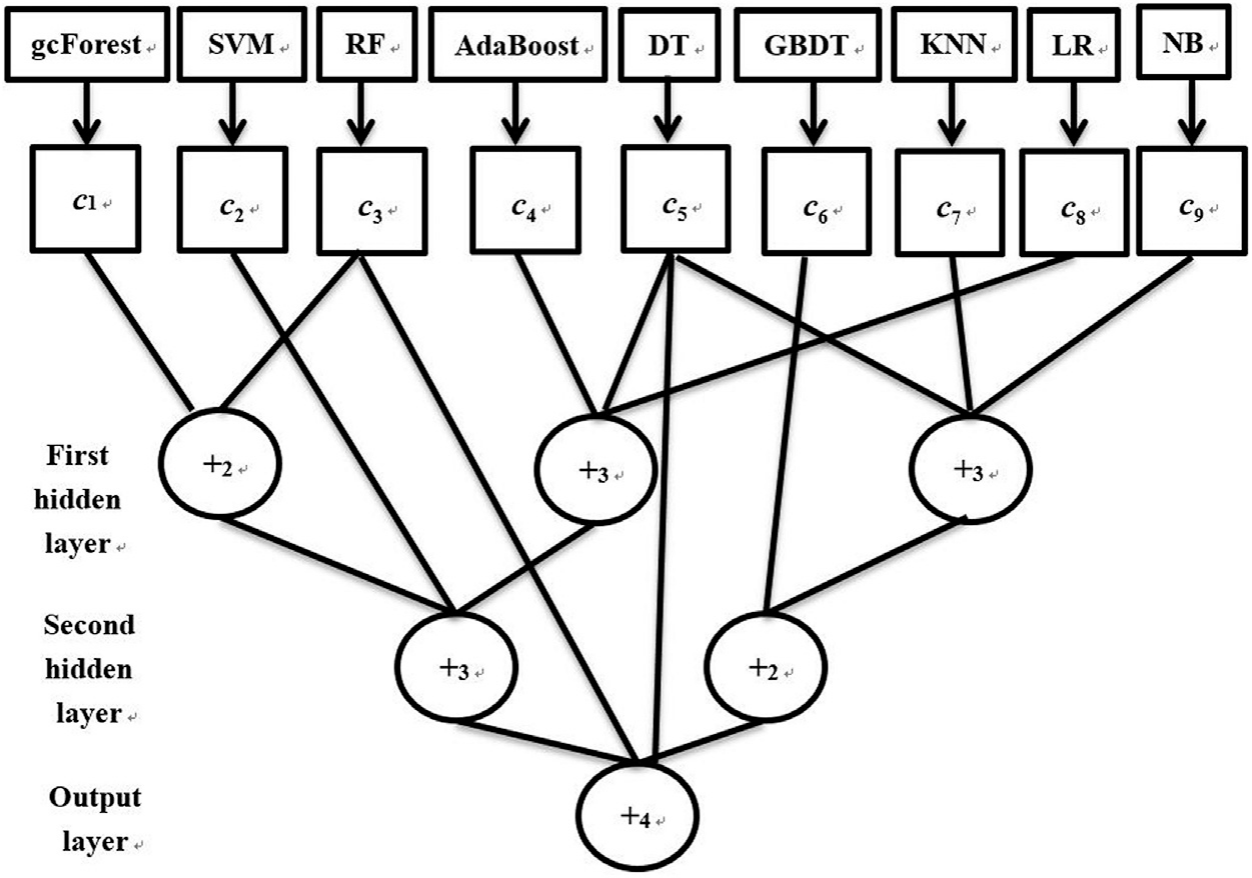
The flowchart of our proposed ensemble method.

To improve the classification performance of a single classifier, a novel ensemble method based on a flexible neural tree (FNT) is proposed. An example of our proposed ensemble method is depicted in Figure 2. From Figure 2, it could be seen that the used base classifiers are gcForest, SVM, RF, AdaBoost, decision tree, GBDT, KNN, logical regression, and naïve Baye, which are introduced in detail in *Classifiers*. Firstly according to the training data, these nine classifiers can output their corresponding confidence level set ( $c=\left(c_{1},c_{2},\ldots ,c_{9}\right)$ ), which is utilized as the input layer of the FNT model. The other hidden layers of the FNT model can be created randomly from operator set ( $F=\left({+}_{2},{+}_{3},\ldots ,{+}_{n}\right)$ ) and variable set ( $T=\left(c_{1},c_{2},\ldots ,c_{9}\right)$ ) (Chen et al., 2006). ${+}_{i}$ denotes a flexible neuron operator, which can be calculated as follows:

$$\left\{ \begin{array}{l} net_{i}=\sum_{j=1}^{i}w_{j}x_{j},\\ o_{i}=f\left(a_{i},b_{i},net_{i}\right)=e^{-{\left(\frac{net_{i}-a_{i}}{b_{i}}\right)}^{2}}. \end{array}\right.$$ (17)

Where f(⋅) is an activation function, $a_{i}$ and $b_{i}$ are the parameters of function, $x_{j}$ is the input variable and $w_{j}$ is the corresponding weight of the input variable.

FNT is a kind of cross-layer neural network, so each hidden layer can contain both operator and variable nodes. Because the structure of the FNT model is not fixed and this model contains many parameters such as $a_{i}$ , $b_{i}$ , and $w_{j}$ , many swarm algorithms have been proposed to search the optimal FNT model by iterations. In this paper, a hybrid evolutionary method based on genetic programming like structure optimization algorithm and simulated annealing was utilized for the training dataset. The detailed algorithms were introduced in another study (Yang et al., 2013).

### Hypertension-Related Activity Drug Identification

**FIGURE 3:**
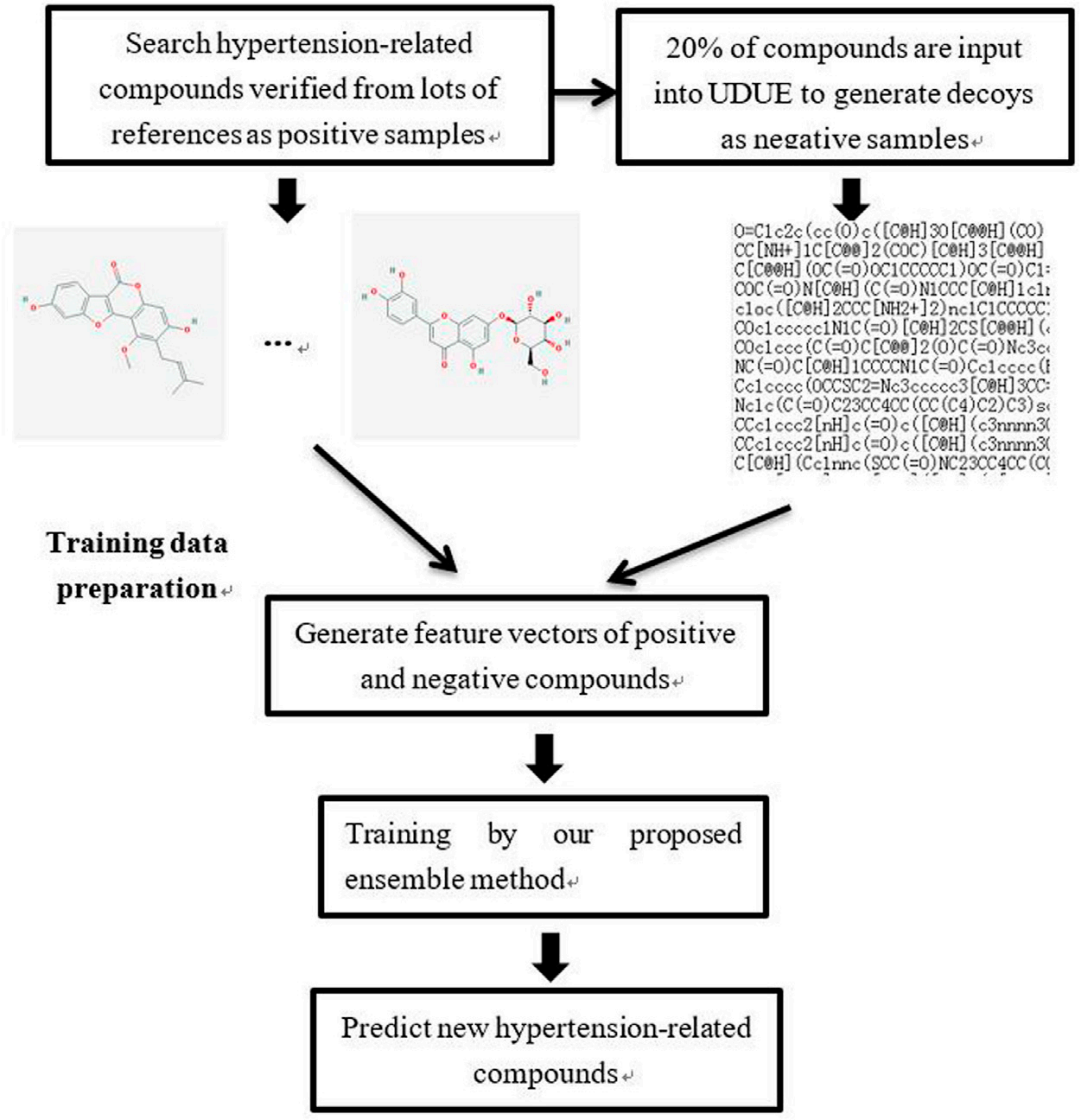
The flowchart of hypertension-related active compound identification.

In order to identify hypertension-related active compounds accurately, an ensemble method based on nine classifiers and a flexible neural tree is proposed. The process of hypertension-related active compounds identification is depicted in Figure 3. A total of 44 important studies were collected by querying the literature database according to two keywords: hypertension and network pharmacology. Through analyzing this literature, many important medicines such as Banxia Baizhu Tianma Tang, Chaihu Longgu Muli Decoction, compound reserpine and triamterene tablets, and Huanglian Jiedu Decoction, were collected and 88 hypertension-related compounds were searched. These important compounds were verified by biology experiments or molecular docking, which were used as positive samples in this paper. To obtain the negative samples, 20% of these compounds were randomly selected and input into the DUD•E website to generate decoys (Mysinger et al., 2012). In total, 264 decoys are selected randomly as negative samples.

The molecular descriptions of positive and negative compounds were extracted to constitute the hypertension-related dataset. With the collected dataset, our proposed ensemble method was fitted to predict other hypertension-related compounds.

## Experiment Results

In this part, the hypertension-related dataset collected is utilized, which contains 88 related compounds and 264 unrelated compounds. AUC, ROC curve, TPR, FRP, Precision, Specificity, and F1 were used to test the performance of our proposed method. In our method, the parameters of nine classifiers were set by default. In FNT, the variable set is defined as $T=\left(c_{1},c_{2},\ldots ,c_{9}\right)$ and the operator set is defined as $F=\left({+}_{2},{+}_{3},{+}_{4},{+}_{5}\right)$ .

**FIGURE 4:**
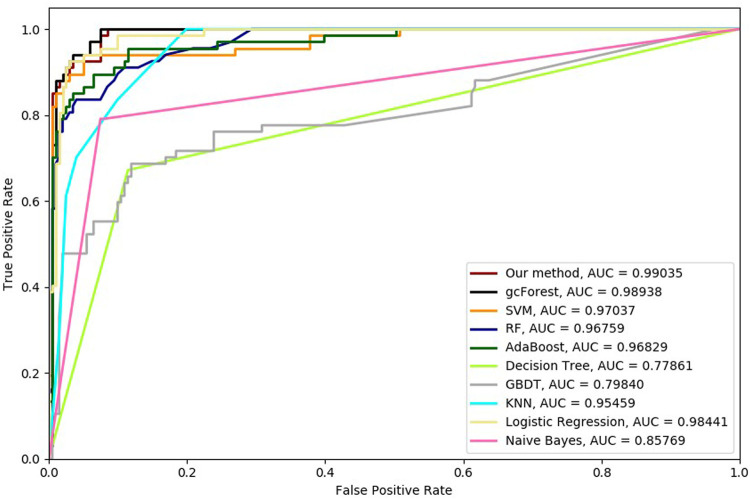
Hypertension-related compound identification performances of ten methods with 2-cross validation methods.

**FIGURE 5:**
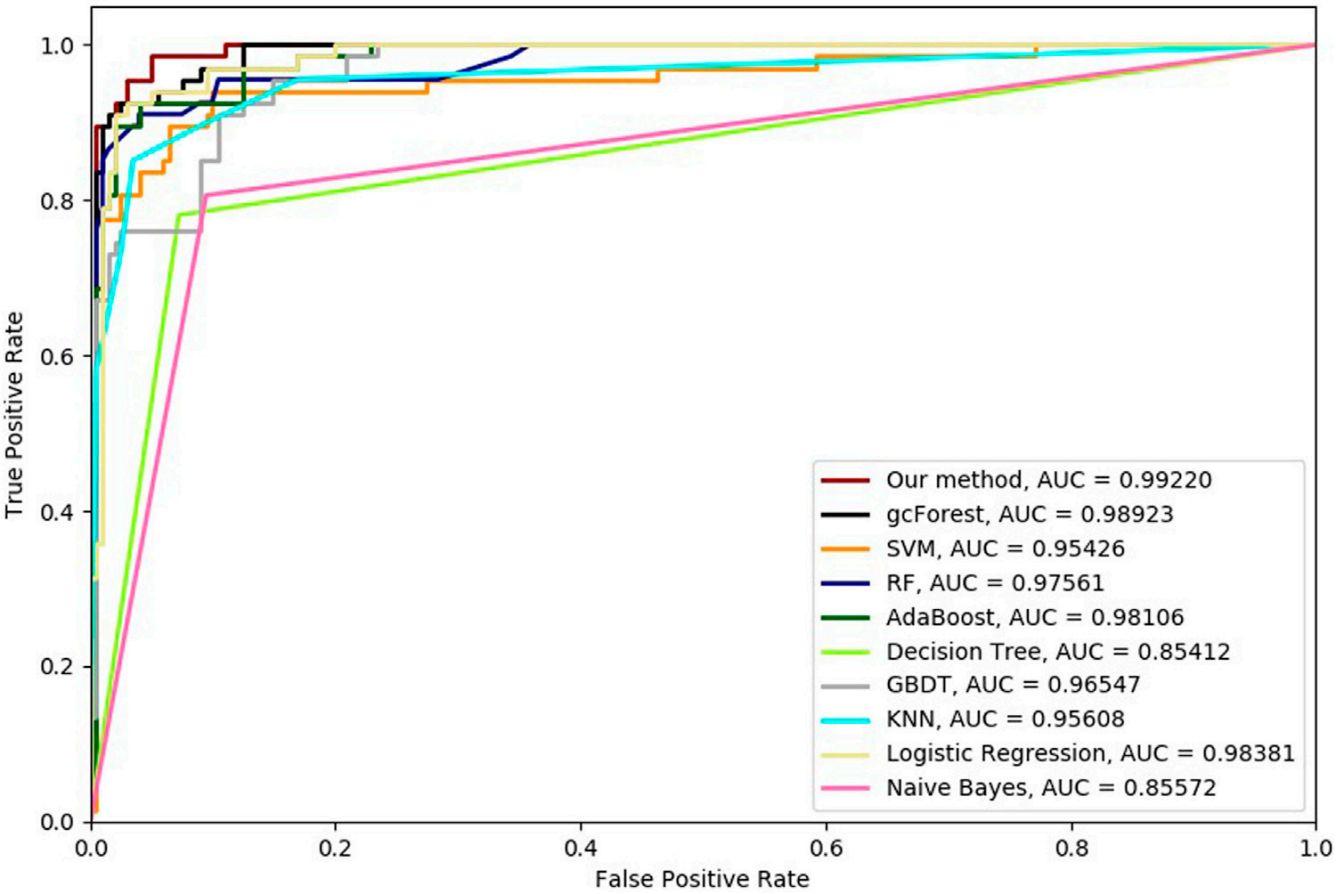
Hypertension-related compound identification performances of ten methods with 4-cross validation methods.

**FIGURE 6:**
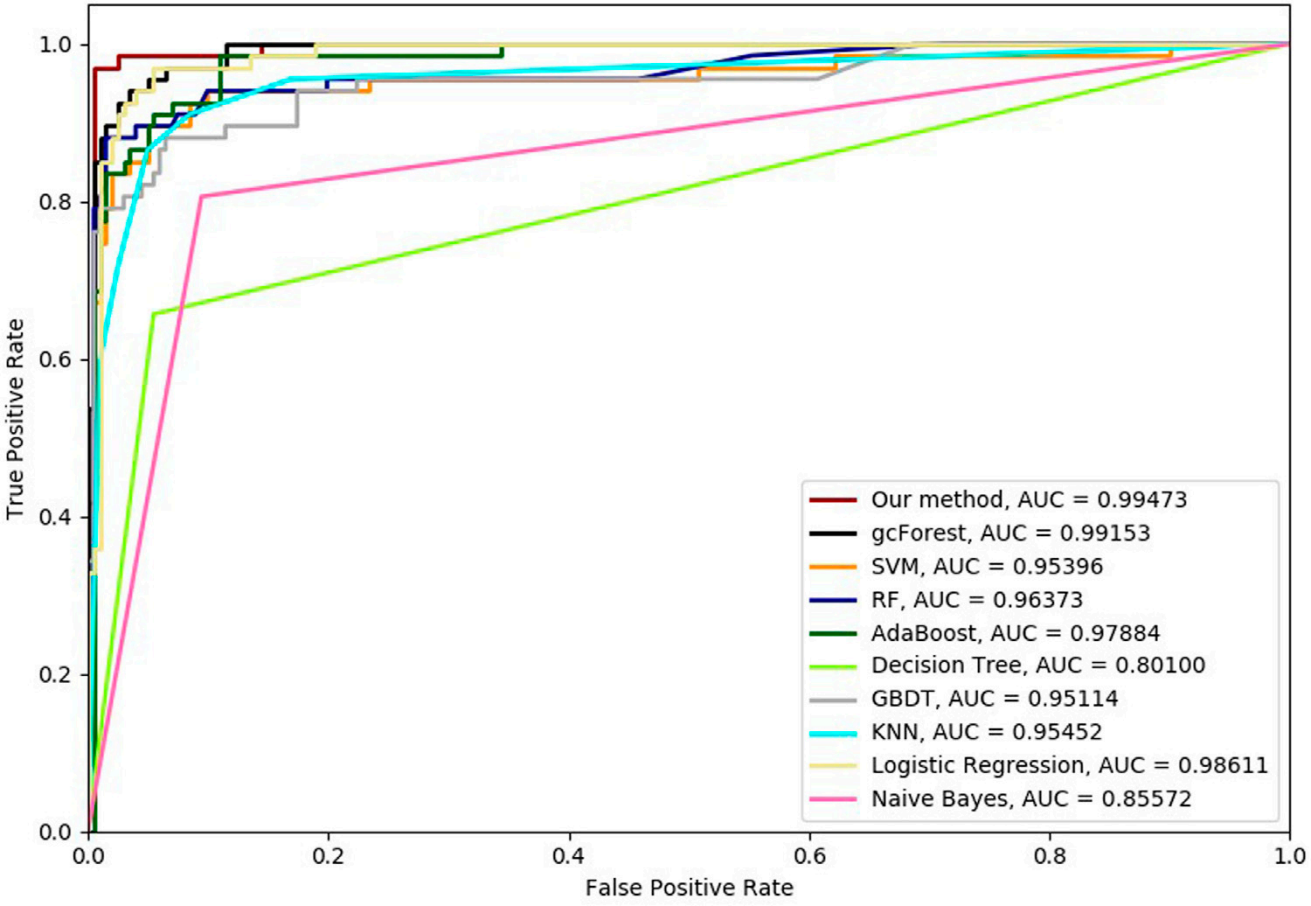
Hypertension-related compound identification performances of ten methods with 6-cross validation methods.

**FIGURE 7:**
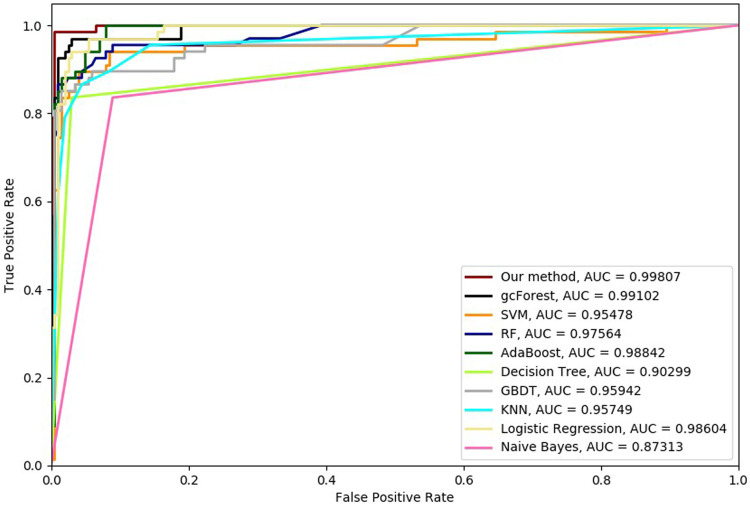
Hypertension-related compound identification performances of ten methods with 8-cross validation methods.

**FIGURE 8:**
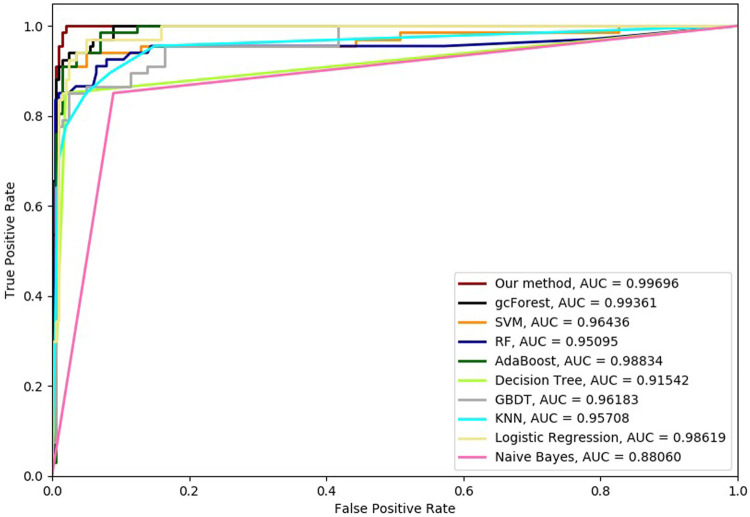
Hypertension-related compound identification performances of ten methods with 10-cross validation methods.

**FIGURE 9:**
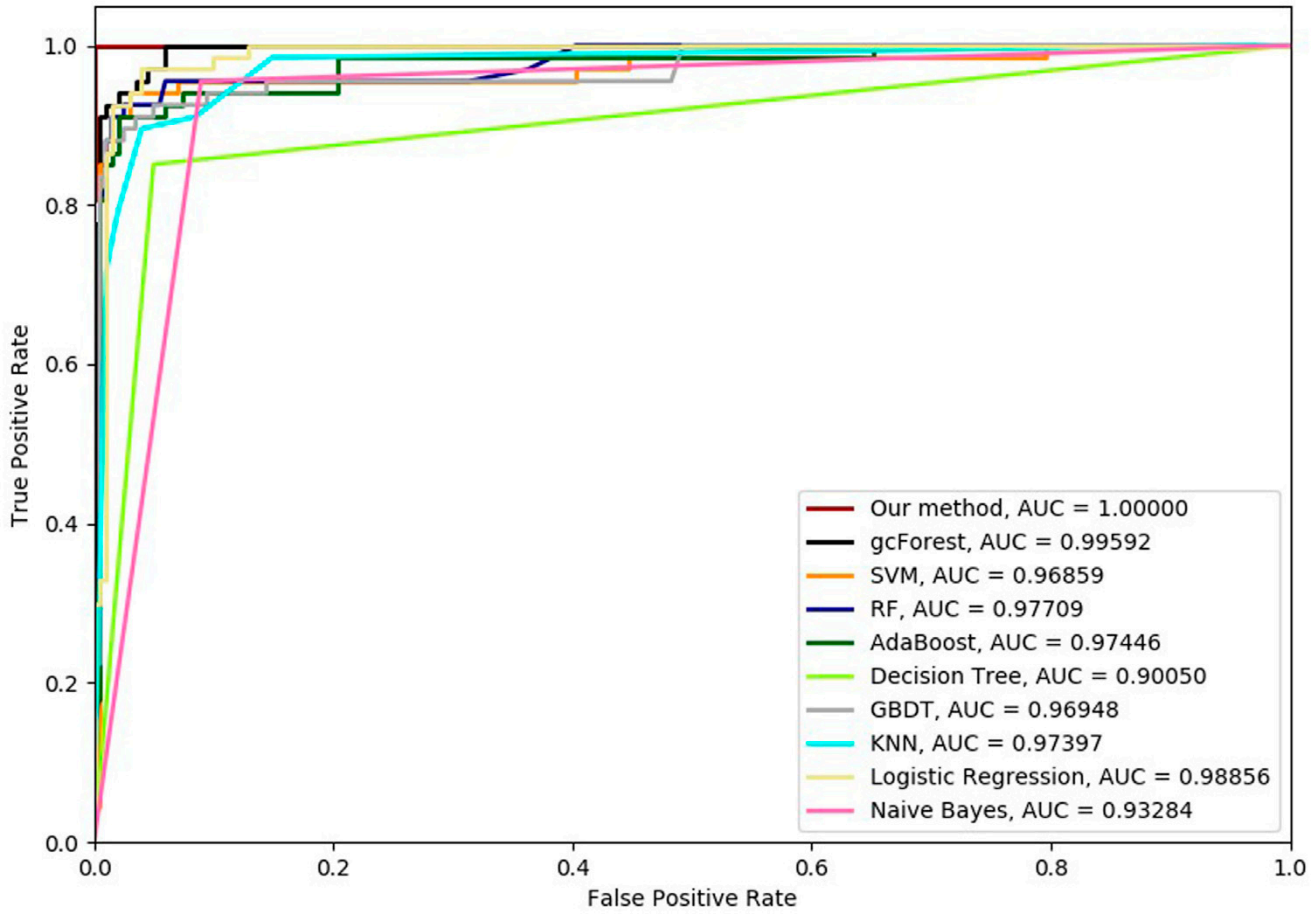
Hypertension-related compound identification performances of ten methods with 15-cross validation methods.

Six cross-validation methods were utilized to validate our proposed method. Nine classifiers were also utilized to identify hypertension-related compounds with the same dataset. The ROC curves and AUC performances with the different cross-validation methods are depicted in Figures 4–9, respectively. From these results, it can be seen that gcForest has the best ROC curves and AUC values among the nine single classifiers. Our proposed ensemble method could perform better than gcForest in terms of ROC and AUC. With 2-cross, 4-cross, 6-cross, 8-cross, 10-cross, and 15-cross validation methods, in terms of AUC, our method is 0.1, 0.3, 0.3, 0.7, 0.3, and 0.4% higher than gcForest, which reveals that our proposed method performs better than nine single classifiers for hypertension-related compound identification.

The TPR, FRP, Precision, Specificity, and F1 performances of the ten methods with the different cross-validation methods are listed in Tables 1–6, respectively. With 2-cross validation and 4-cross validation methods, LR could obtain the highest TPR performances, which shows that LR could identify more true hypertension-related compounds. For Table 1, RF and SVM have the best FPR performance, which shows that these two methods could identify less non-related compounds as related ones. SVM also has the highest Precision and Specificity performances among the ten methods. For Table 2, RF has the best FPR, Precision, and Specificity performances. Our method performed best in terms of F1, which reveals that it could identify hypertension-related compounds more accurately overall. With 6-cross validation, 8-cross validation, 10-cross validation, and 15-cross validation methods, our methods perform best among ten methods in terms of TPR, FRP, Precision, Specificity, and F1, except that RF has the lowest performance with 4-cross validation methods. The results show that our proposed ensemble method could identify more true hypertension-related and hypertension-unrelated compounds than the other nine single classifiers.

**TABLE 1:** Classification performances of ten methods with 2-cross validation methods.

	TPR	FRP	Precision	Specificity	F1
Our method	0.880597	0.019900	0.936508	0.980100	0.907692
gcForest	0.940299	0.054726	0.851351	0.945274	0.893617
AdaBoost	0.791045	0.014925	0.946429	0.985075	0.861789
Decision Tree	0.671642	0.114428	0.661765	0.885572	0.666667
GBDT	0.61194	0.104478	0.66129	0.895522	0.635659
KNN	0.701493	0.039801	0.854545	0.960199	0.770492
LR	0.985075	0.199005	0.622642	0.800995	0.763006
Naive Bayes	0.791045	0.074627	0.779412	0.925373	0.785185
RF	0.671642	0.00995	0.957447	0.99005	0.789474
SVM	0.850746	0.00995	0.966102	0.99005	0.904762

**TABLE 2:** Classification performances of ten methods with 4-cross validation methods.

	TPR	FRP	Precision	Specificity	F1
Our method	0.895522	0.014925	0.952381	0.985075	0.923077
gcForest	0.925373	0.039801	0.885714	0.960199	0.905109
AdaBoost	0.835821	0.0199	0.933333	0.9801	0.88189
Decision Tree	0.686567	0.039801	0.851852	0.960199	0.760331
GBDT	0.671642	0.00995	0.957447	0.99005	0.789474
KNN	0.850746	0.034826	0.890625	0.965174	0.870229
LR	0.940299	0.074627	0.807692	0.925373	0.868966
Naive Bayes	0.80597	0.094527	0.739726	0.905473	0.771429
RF	0.791045	0.00995	0.963636	0.99005	0.868852
SVM	0.776119	0.024876	0.912281	0.975124	0.83871

**TABLE 3:** Classification performances of ten methods with 6-cross validation methods.

	TPR	FRP	Precision	Specificity	F1
Our method	0.955224	0.004975	0.984615	0.995025	0.969697
gcForest	0.925373	0.024876	0.925373	0.975124	0.925373
AdaBoost	0.835821	0.0199	0.933333	0.9801	0.88189
Decision Tree	0.656716	0.054726	0.8	0.945274	0.721311
GBDT	0.791045	0.00995	0.963636	0.99005	0.868852
KNN	0.865672	0.049751	0.852941	0.950249	0.859259
LR	0.940299	0.049751	0.863014	0.950249	0.9
Naive Bayes	0.80597	0.094527	0.739726	0.905473	0.771429
RF	0.820896	0.014925	0.948276	0.985075	0.88
SVM	0.791045	0.014925	0.946429	0.985075	0.861789

**TABLE 4:** Classification performances of ten methods with 8-cross validation methods.

	TPR	FRP	Precision	Specificity	F1
Our method	0.970149	0.004975	0.984848	0.995025	0.977444
gcForest	0.940299	0.0199	0.940299	0.9801	0.940299
AdaBoost	0.850746	0.014925	0.95	0.985075	0.897638
Decision Tree	0.835821	0.029851	0.903226	0.970149	0.868217
GBDT	0.80597	0.004975	0.981818	0.995025	0.885246
KNN	0.865672	0.044776	0.865672	0.955224	0.865672
LR	0.940299	0.044776	0.875	0.955224	0.906475
Naive Bayes	0.835821	0.089552	0.756757	0.910448	0.794326
RF	0.835821	0.00995	0.965517	0.99005	0.896
SVM	0.791045	0.014925	0.946429	0.985075	0.861789

**TABLE 5:** Classification performances of ten methods with 10-cross validation methods.

	TPR	FRP	Precision	Specificity	F1
Our method	0.955224	0.014925	0.955224	0.985075	0.955224
gcForest	0.925373	0.0199	0.939394	0.9801	0.932331
AdaBoost	0.850746	0.014925	0.95	0.985075	0.897638
Decision Tree	0.850746	0.0199	0.934426	0.9801	0.890625
GBDT	0.776119	0.014925	0.945455	0.985075	0.852459
KNN	0.850746	0.049751	0.850746	0.950249	0.850746
LR	0.940299	0.044776	0.875	0.955224	0.906475
Naive Bayes	0.850746	0.089552	0.76	0.910448	0.802817
RF	0.820896	0.004975	0.982143	0.995025	0.894309
SVM	0.880597	0.014925	0.951613	0.985075	0.914729

**TABLE 6:** Classification performances of ten methods with 15-cross validation methods.

	TPR	FRP	Precision	Specificity	F1
Our method	0.955224	0	1	1	0.977099
gcForest	0.940299	0.0199	0.940299	0.9801	0.940299
AdaBoost	0.880597	0.0199	0.936508	0.9801	0.907692
Decision Tree	0.850746	0.049751	0.850746	0.950249	0.850746
GBDT	0.835821	0.00995	0.965517	0.99005	0.896
KNN	0.895522	0.039801	0.882353	0.960199	0.888889
LR	0.940299	0.034826	0.9	0.965174	0.919708
Naive Bayes	0.955224	0.089552	0.780488	0.910448	0.85906
RF	0.850746	0.00995	0.966102	0.99005	0.904762
SVM	0.880597	0.014925	0.951613	0.985075	0.914729

## Discussion

**FIGURE 10:**
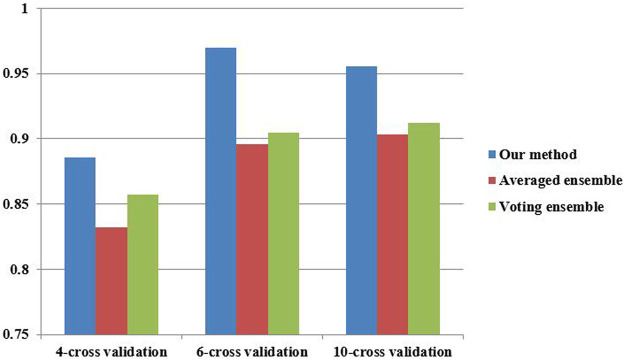
F1 performances of hypertension-related compound by three ensemble methods.

**FIGURE 11:**
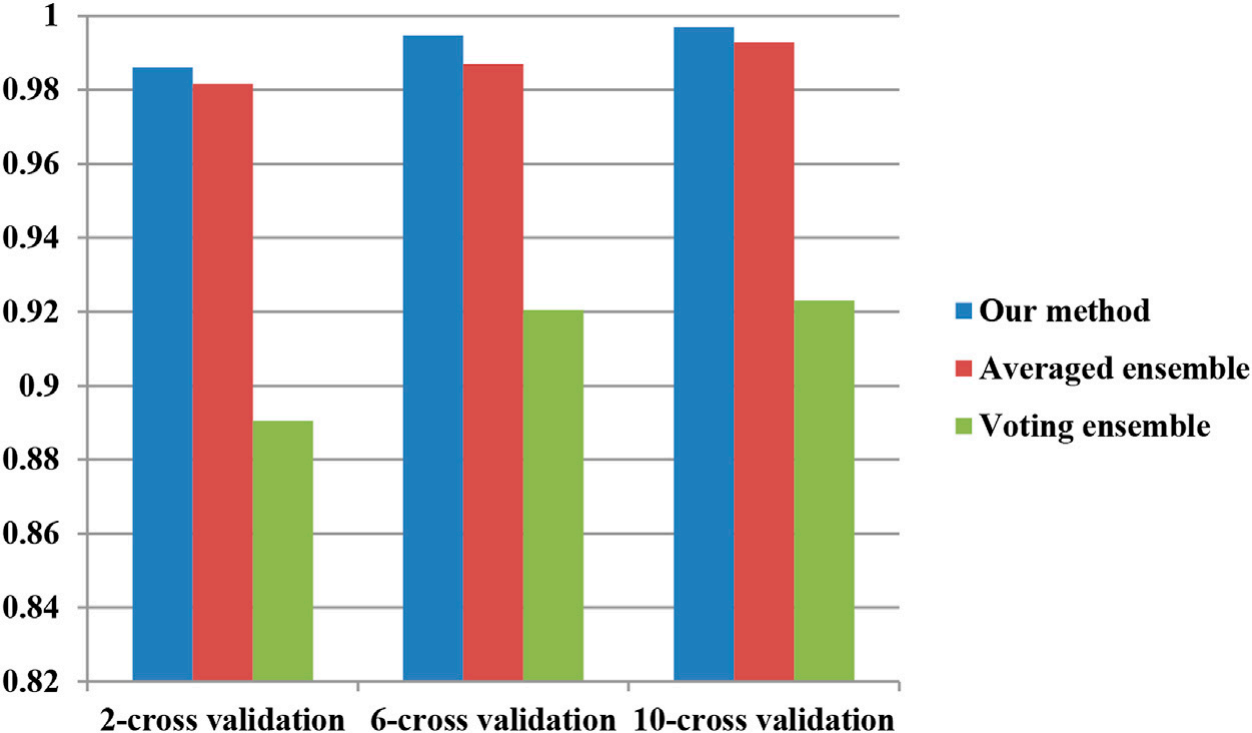
AUC performances of hypertension-related compound by three ensemble methods.

To investigate the performance of our proposed ensemble further, two classical ensemble methods (averaged ensemble and voting ensemble) were also utilized to infer hypertension-related compounds. The F1 and AUC performances of the hypertension-related compounds by three ensemble methods are depicted in Figure 10 and Figure 11, respectively. From Figures 10, 11, it can be seen that our proposed ensemble method obtained better F1 and AUC performances than averaged and voting ensemble methods, which also shows that our method could identify hypertension-related compounds more accurately than the other two classical ensemble methods.

## Conclusion

To identify hypertension-related closely active compounds, this paper proposed a novel ensemble method based on a flexible neural tree and nine classifiers. In our method, the classification results of nine single classifiers was utilized as the input vector of the flexible neural tree. An FNT model was utilized as a nonlinear ensemble method to identify hypertension-related drug activity. A hybrid evolutionary method based on genetic programming like structure optimization algorithm and simulated annealing is proposed to evolve the FNT model. In order to test the performance of our proposed ensemble method, data were extracted from hypertension-unrelated and hypertension-related compounds collected from up-to-date literature. By the different cross-validation methods, our proposed method obtained better ROC curves and AUC values than nine other single classifiers. Our proposed method also performs better than other single classifiers in terms of TPR, FRP, Precision, Specificity, and F1 in most cases. We also compare our proposed ensemble method with the averaged and voting ensemble methods. The results reveal that our method could identify hypertension-related compounds more accurately than the two classical ensemble methods.

## Data Availability

The original contributions presented in the study are included in the article/Supplementary Material, further inquiries can be directed to the corresponding author.
